# Optimized design for illusion device by genetic algorithm

**DOI:** 10.1038/s41598-021-99055-9

**Published:** 2021-09-30

**Authors:** Zhenzhong Yu, Zhong Yang, Yan Zhang, Yizhi Wang, Xingliu Hu, Xiaomin Tian

**Affiliations:** 1 College of Science, Nanjing University of Aeronautics and Astronautics, Nanjing, 210016 China; 2 School of Intelligence Science and Control Engineering, Jinling Institute of Technology, Nanjing, 211169 China

**Keywords:** Optics and photonics, Physics

## Abstract

The illusion device developed from the scattering cancellation employs very simple homogeneous and isotropic materials, but this device is only valid for electrically small objects. In this paper, we prove that the illusion device optimized by genetic algorithm can be applied to large-scale occasions. For an electrically small target, an optimized core–shell illusion device can achieve better illusion effect than the analytical design based on the scattering cancellation. With the increase of the device size, the ability of the single-layered shell to manipulate the scattering is very limited. For a moderate-size target, two optimized multi-layered examples are presented: one is to make a dielectric cylinder appear as another dielectric target, and the other is to make a conducting cylinder behave like a double-negative-material target. The full-wave simulations are carried out to visualize the similar field distributions of the target and the optimized multi-layered design. This optimized design greatly widens the size application range of the illusion device and can also improve the illusion performance with simple material parameters.

## Introduction

Transformation optics (TO), a mathematical tool to calculate the material parameters based on form-invariant transformations of Maxwell’s equations, has been employed to design a wide variety of functional optical devices including invisibility cloaks^1–3^, illusion optical devices^4–10^, and so on.

Illusion optical devices are capable of reshaping the image size, shape, location, or constitutive parameters of an object, which may have potential applications in the military and other fields. The invisibility cloak can be thought of as a special case of optical illusion, in which the scattering of an object is dramatically reduced to imitate that of the free space. Lai et al. first proposed the concept of illusion optics which makes an object look like another one^4^. Various kinds of illusion devices have been designed by using TO, such as superscatterers, PEC-dielectric conversion devices, shrinking devices, overlapping illusion, ghost illusion and so on^5–10^. In addition, the concept of optical illusion has also been further extended to many physical fields, such as electrostatics, acoustics, and thermodynamics^11–14^. However, the materials of these intriguing devices are often highly complex, because the material parameters are inhomogeneous, anisotropic, and even negative refractive index. These are the main difficulties that restrict the practical application of these illusion devices. Consequently, a sequence of approaches to simplify the fabrication have been proposed, such as conformal transformation optics (CTO), linear coordinate transformation (LCT), method of the wave vector domain modification (WVDM), and scattering cancellation^15–24^. The illusion devices achieved by the methods of CTO and WVDM can be implemented by using normal dielectrics, but the constitutive parameters are inhomogeneous which will lead to difficulty in fabrication. Linear coordinate transformation has been utilized to avoid the inhomogeneous parameters, but the calculated anisotropic transformation material is still a challenge for practical realization. According to the effective medium theory, a single anisotropic material can be replaced by two alternating thin isotropic layers^25^. This theory is more accurate when the thickness of each layer is much less than the wavelength and the number of the layers is large enough, which will greatly increase the complexity of realization.

Another approach to achieving the electromagnetic illusion is by means of the scattering cancellation mechanism first proposed by Alu and originally used to design the invisibility cloak^18–20^. This design method is realized by analyzing the scattering coefficients under the quasi-static condition, which limits the applications of the illusion device to the size range far less than the wavelength. In 2015, F. Yang et al. generalized the scattering cancellation for the design of a spherical illusion device that a core–shell structure with isotropic and homogeneous materials can be equivalent to a homogeneous sphere (electrically small) with a different outer radius^21^. Later, the illusions of inhomogeneous and anisotropic cylinder (sphere) were proposed that scattering distribution of the inhomogeneous cylinder (sphere) can be controlled by covering a well-designed shell such that its scattering is similar to that of an isotropic cylinder (sphere)^22,23^. In our previous research, we studied a cylindrical core–shell illusion device composed of isotropic and homogeneous materials and calculated the formula for the illusion condition in the quasi-static approximation^24^. Contour maps for the formula were utilized to visualize the relationship between the parameters of the target and the shell^24^. Although the illusion device designed by scattering cancellation commonly uses an anisotropic or even isotropic coating that is easy to fabricate, this device can produce a good illusion only if its size is much smaller than the wavelength. Therefore, the illusion device based on scattering cancellation is severely restricted in practical applications.

For an object with its dimension comparable to wavelength, the higher-order scattering modes become evident, which may make the performance of illusion device based on the analytical theory of the scattering cancellation less effective^25^. Similar problems have also appeared in the cloaking design. In the study of the invisibility cloak, we have constructed a concentric multi-layered shell for different-sized concealed targets by optimizing the material and geometrical parameters of each covering layer^25,26^. To our best knowledge, optimized design of illusion devices with multi-layered homogeneous isotropic materials has not yet been studied. In this paper, firstly we optimize a cylindrical core–shell structure to make its scattering equivalent to that of a homogeneous cylinder through genetic algorithm (GA). For an electrically small target whose size is one tenth of the wavelength, the illusion effect of this optimized structure is much better than that of a scattering-cancellation illusion device. With the increase of the device size, a multi-layered illusion device is adopted to increase the freedom of optimized parameters and has better illusion effect than the simple core–shell structure. Subsequently, two examples are presented: one is to make a dielectric object appear as a larger target with different permittivity, and the other is to make a conducting cylinder behave like a target with a double negative material. There are at least two advantages of this optimized design. The first one is that this optimized illusion device is achieved with several layers of homogeneous isotropic materials, which is easy for practical implementation. The second is that compared with the scattering cancellation method, this optimized design can be applied to large-scale occasions and produce better illusion effect. The comparison between this optimization method and other design methods is shown in Table 1.

**Table 1:** Comparison of the optimization method proposed in this paper with other illusion design methods.

Characteristics	CTO/WVDM	LCT	Scattering cancellation	Optimization method
Material	Inhomogeneous, nonmagnetic, and isotropic	Homogeneous, and anisotropic	Homogeneous, isotropic or anisotropic	Homogeneous, and isotropic
Size	Applicable to any size	Applicable to any size	Much smaller than wavelength	Applicable to large size

## Results

**Figure 1:**
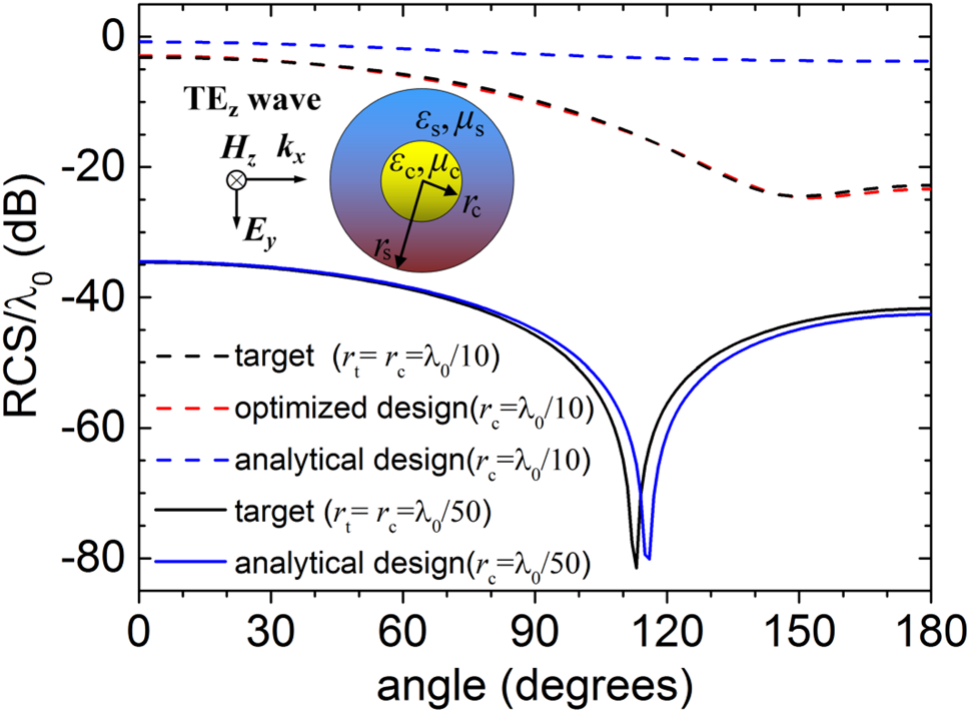
The scattering widths normalized by wavelength for the optimized design and the analytical design. Two different radii of the core are considered, namely *r*_c_ = *λ*_0_/10 (dashed lines) and *r*_c_ = *λ*_0_/50 (solid lines). We assume that the radii of the core and the target are equal, that is *r*_t_ = *r*_c_. The constitutive parameters of the core and the target are (3*ε*_0_, 3*μ*_0_) and (5*ε*_0_, 1.5*μ*_0_) respectively. The inset shows that a TE_Z_ plane wave is normally incident on a cylindrical core coated by a homogeneous isotropic shell.

As the first example, we consider an electrically small cylindrical core–shell illusion device made of isotropic materials which is placed in the free space with constitutive parameters (*ε*_0_, *μ*_0_), shown in the inset of Fig. 1. The radius, permittivity and permeability of the core–shell structure are denoted as (*r*_*c*_, *ε*_*c*_, *μ*_*c*_) and (*r*_*s*_, *ε*_*s*_, *μ*_*s*_), respectively. This core–shell structure with elaborate design can be disguised as a target cylinder with parameters (*r*_*t*_, *ε*_*t*_, *μ*_*t*_). For simplicity, a TE_*z*_ polarized plane wave is taken into consideration, and only the component of magnetic field in the cylinder axis (*z*-direction) is non-zero. According to the Mie theory, the scattered magnetic field from the multi-layers or the bare target cylinder can be represented in the cylindrical coordinates (*r*, *θ*, *z*) by the sum of Hankel functions of the second kind as^27^

1 $$ H_{z}^{sca} (r,\theta ) = \sum\limits_{n = - \infty }^{ + \infty } {c_{n} H_{n}^{(2)} (k_{0} r)e^{jn\theta } } $$

where $k_{0} { = }\omega \sqrt {\mu_{0} \varepsilon_{0} }$ represents the wave number in the free space, and *c*_*n*_ represents the scattering coefficient which can be determined by applying the boundary condition of each layer. In order to compare the far-field scatterings from the illusion device and the target object, it is necessary to calculate the bistatic scattering width which is usually represented by^27^

2 $$ \sigma (\theta ) = \frac{4}{{k_{0} }}\left| {\sum\limits_{n = - \infty }^{{{ + }\infty }} {c_{n} j^{n} e^{jn\theta } } } \right|^{2} $$

When the radius of the cylindrical structure is much smaller than the working wavelength, the scattering is mainly composed of the *n* = 0 and $n = \pm 1$ scattering terms. To achieve a good illusion performance in the quasi-static condition, the scattering coefficients $c_{n}^{core - shell}$ for the core–shell illusion device and the coefficients $c_{n}^{{t{\text{arge}}t}}$ for the target cylinder should satisfy the following equation

3 $$ c_{n}^{core - shell} = c_{n}^{{{\text{target}}}} ,\quad \left| n \right| = 0,\;1. $$

Optimization procedure based on GA, once used to optimize the multilayered cloak, is applied to achieve the core–shell illusion device. The fitness function *f* in the GA can be chosen as the accumulation of the difference between the scattering coefficients $c_{n}^{core - shell}$ and $c_{n}^{{t{\text{arge}}t}}$, which is described as

4 $$ f = \sum\limits_{n = - 1}^{1} {\left| {c_{n}^{core - shell} - c_{n}^{{{\text{target}}}} } \right|} $$

**Figure 2:**
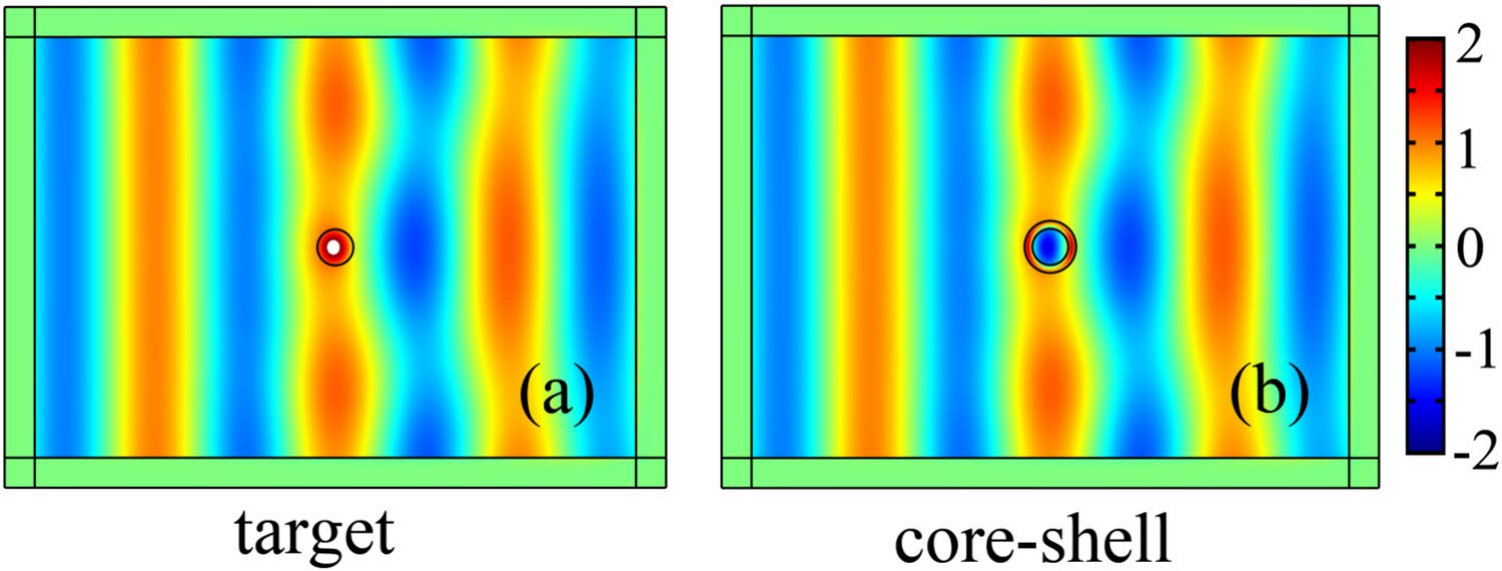
A TE_z_ polarized plane wave with unit magnitude is incident horizontally from the left side. The *z*-directed magnetic field distributions of the target with parameters *r*_*t*_ = λ_0_**/**10, *ε*_*t*_ = 5*ε*_0_ and *μ*_*t*_ = 1.5*μ*_0_ **(a)** and the optimized core–shell illusion device **(b)** are simulated by using COMSOL Multiphysics. The parameters of the core are *r*_*c*_ = λ_0_**/**10, *ε*_*c*_ = 3*ε*_0_ and *μ*_*c*_ = 3*μ*_0_ and those of the shell are *r*_*s*_ = 1.44*r*_c_, *ε*_*s*_ = 15.45*ε*_0_, and *μ*_*s*_ = 6.93*μ*_0_. The magnitudes out of the colour bar range are represented by white area.

Setting the parameters of the core (*r*_*c*_, *ε*_*c*_, *μ*_*c*_) and the target (*r*_*t*_, *ε*_*t*_, *μ*_*t*_) in advance, we optimize the parameters of the covering shell (*r*_*s*_, *ε*_*s*_, *μ*_*s*_) to achieve the equivalent scattering. In this case, the fitness function *f*(*r*_*s*_, *ε*_*s*_, *μ*_*s*_) is a function of the shell parameters. GA searches for the optimal shell parameters by making the fitness function approach to the minimum value. In our previous study^24^, the design of the core–shell illusion device is based on the analytical formula method which is only applicable to the case that the device size is far less than the wavelength *λ*_0_, often less than *λ*_0_/15. To obtain a very similar scattering distribution, the size of the illusion device should be even less than *λ*_0_/50. Compared with the analytical design, the optimized design based on GA has more degrees of freedom, and greatly widens the size application range of the illusion device. In this section, the shell parameters of the illusion device are designed by the analytical method and the optimization approach, respectively. We consider that the constitutive parameters of the central core are (3*ε*_0_, 3*μ*_0_), those of the target cylinder (5*ε*_0_, 1.5*μ*_0_), and assume that the radii of core and target are equal, namely the radius ratio γ_*t*_ = *r*_t_/*r*_c_ = 1. In our previous analytical design, contour maps were used to visualize the corresponding relationship between the parameters of the target and the core–shell structure^24^. It can be concluded from the contour maps that when the thickness is adjustable the shell has many suitable parameters. Under the above parameters of the core and the target, the permittivity of the shell can be satisfied in the regions of {ε_s_/ε_0_ > 1} and {0 > ε_s_/ε_0_ > −4.3}. If a shell of moderate thickness is selected with *r*_s_ = 1.44*r*_c_, the constitutive parameters of the shell can be calculated as (1.342*ε*_0_, -0.3972*μ*_0_) from the analytical formula^24^. Next, we use the genetic algorithm to optimize the design, assuming that the radius of the core cylinder is *r*_c_ = *λ*_0_/10. Considering the practical constraints on the constitutive parameters of the shell, we limit the search space of the relative parameters in the optimization process to between 1 and 20. The optimized results for the shell parameters (*r*_*s*_, *ε*_*s*_, *μ*_*s*_) are (1.44*r*_c_, 15.45*ε*_0_, 6.93*μ*_0_) by minimizing the fitness function. Figure 1 shows the far-field scattering widths when a TE_*z*_-polarized plane wave is normally incident onto the optimized device and the analytical device respectively. Three dashed lines in the figure correspond to the case that the radius of the core cylinder is *r*_c_ = *λ*_0_/10, in which the black dashed line is the RCS curve of the target cylinder, the blue dashed line is the RCS of the core–shell structure based on the analytical formula and the red dashed line is the RCS of the optimized illusion device. It can be found that the optimized illusion device has a similar scattering distribution to that of the target cylinder. However, the scattering of analytical illusion device is significantly different because the analytical design is only applicable when the device size is much smaller than the wavelength. For example, when the size of the target is *r*_*t*_ = *λ*_0_/50, RCS of the analytical illusion device is very close to that of the target, shown by solid lines in Fig. 1. 2D full-wave EM simulations with the COMSOL Multiphysics, a commercial finite-element analysis package, are carried out to validate the illusion effect. Throughout this paper, the calculation domain is bounded by perfectly matched layers, and a TE_z_ unit plane wave (with magnetic field normal to the *x*–*y* plane) impinges horizontally from the left side. In order to facilitate the comparison of the field distributions, we limit the magnitudes of the magnetic field display to between -2 and 2, and the values outside the range are indicated by white areas. When the radius of the core cylinder is *r*_c_ = *λ*_0_/10, both the target and the optimized core–shell illusion device are simulated. The total magnetic field simulations of the target and the optimized device, shown in Fig. 2a,b, have nearly the same distribution in the exterior region, which verifies the effectiveness of the optimized design for the small target.

The above results show that the optimized design of the core–shell illusion device is applicable to the case that the size of the device is far less than the wavelength. However, with the increase of the device size, more scattering terms become obvious, and the ability of the single-layered shell to manipulate the scattering is very limited. A multilayered structure is needed to increase the freedom of optimized parameters, so that the multilayered illusion device and the target cylinder may have almost the same scattering coefficients, namely

5 $$ c_{n}^{multilayer} = c_{n}^{{{\text{target}}}} ,\quad \left| n \right| = 0,1,2,3 \cdots $$

We still use the optimization method of GA to design the multilayered illusion device. The fitness function *f* in the optimization can be chosen as the sum of the difference of all scattering coefficients, which is described as

6 $$ f = \sum\limits_{n = - \infty }^{ + \infty } {\left| {c_{n}^{multilayer} - c_{n}^{{{\text{target}}}} } \right|} $$

**Figure 3:**
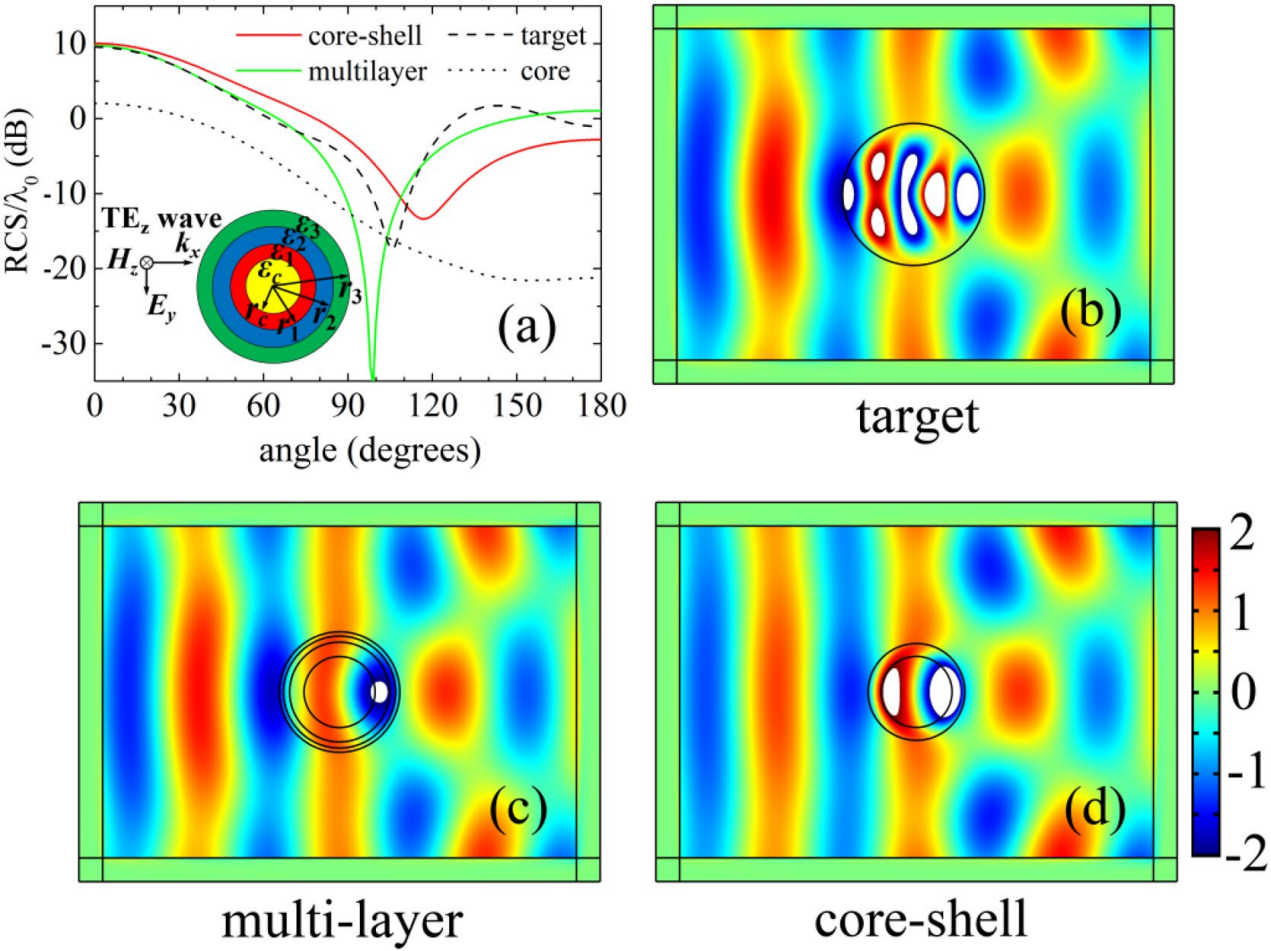
**(a)** The scattering widths normalized by wavelength for the target cylinder with radius *r*_*t*_ = λ_0_**/**2 (dashed black line), the core cylinder with radius *r*_*c*_ = λ_0_**/**4 (dotted black line), the optimized multi-layered design (solid green line), and the optimized core–shell design (solid red line). The inset shows that a core cylinder coated by three layers of homogeneous and isotropic materials. The magnetic field distributions of the target **(b)**, the optimized multi-layered illusion device **(c)**, and the optimized core–shell illusion device **(d)** are simulated by using COMSOL Multiphysics. The magnitudes out of the colour bar range are represented by white area.

As a second optimized example, we consider that the constitutive parameters of the core and the target are (2*ε*_0_, *μ*_0_) and (6*ε*_0_, *μ*_0_), respectively. The radii of the core and the target are different, choosing *r*_*c*_ = λ_0_**/**4 and *r*_*t*_ = λ_0_**/**2. A 3-layer nonmagnetic shell made of ordinary dielectric materials is used to cover the core cylinder, shown in the inset of Fig. 3a. The permittivities and the outer radii of the 3-layer shell, namely $\left\{ {\varepsilon_{1} ,\;\varepsilon_{2} ,\;\varepsilon_{3} ,\;r_{1} ,\;r_{2} ,\;r_{3} } \right\}$, are optimized by GA. Each outer radius is limited to a certain range to ensure a moderate-size shell, and the relative permittivity is constrained between 1 and 20. The optimized results for the 3-layer shell are *ε*_1_ = 1.211*ε*_0_, *ε*_2_ = 3.086*ε*_0_, *ε*_3_ = 6.135*ε*_0_, *r*_1_ = 0.3513λ_0_, *r*_2_ = 0.394λ_0_ and *r*_3_ = 0.424λ_0_. For comparison, a single-layered core–shell illusion device is also optimized with the shell parameters *ε*_s_ = 5.3*ε*_0_ and *r*_s_ = 0.3407λ_0_. In order to compare the omnidirectional scattering characteristics, the far-field normalized scattering widths of the core, target, the optimized core–shell illusion device and the optimized 3-layer device are also investigated, shown in Fig. 3a. It is obviously that the scattering distributions of the core and the target are quite different, but after optimization the scattering distributions of the core–shell device and the 3-layer device are similar to that of the target. Moreover, as for most angles, the scattering width of the optimized 3-layer is closer to that of the target, except for a small angle range around the 100 degrees. Full-wave electromagnetic simulations are also carried out to visualize the illusion effect of the optimized multi-layer and that of the core–shell structure shown in Fig. 3c,d. For comparison, simulation of the target is also conducted, shown in Fig. 3b. It can be observed that the total magnetic field distributions of the target and the optimized multi-layer are almost the same in the outer region. For the optimized core–shell structure, the backscattering is obviously smaller than that of the target. The above results indicate that the optimized 3-layer illusion device can provide more degrees of freedom to achieve a better illusion effect.

**Figure 4:**
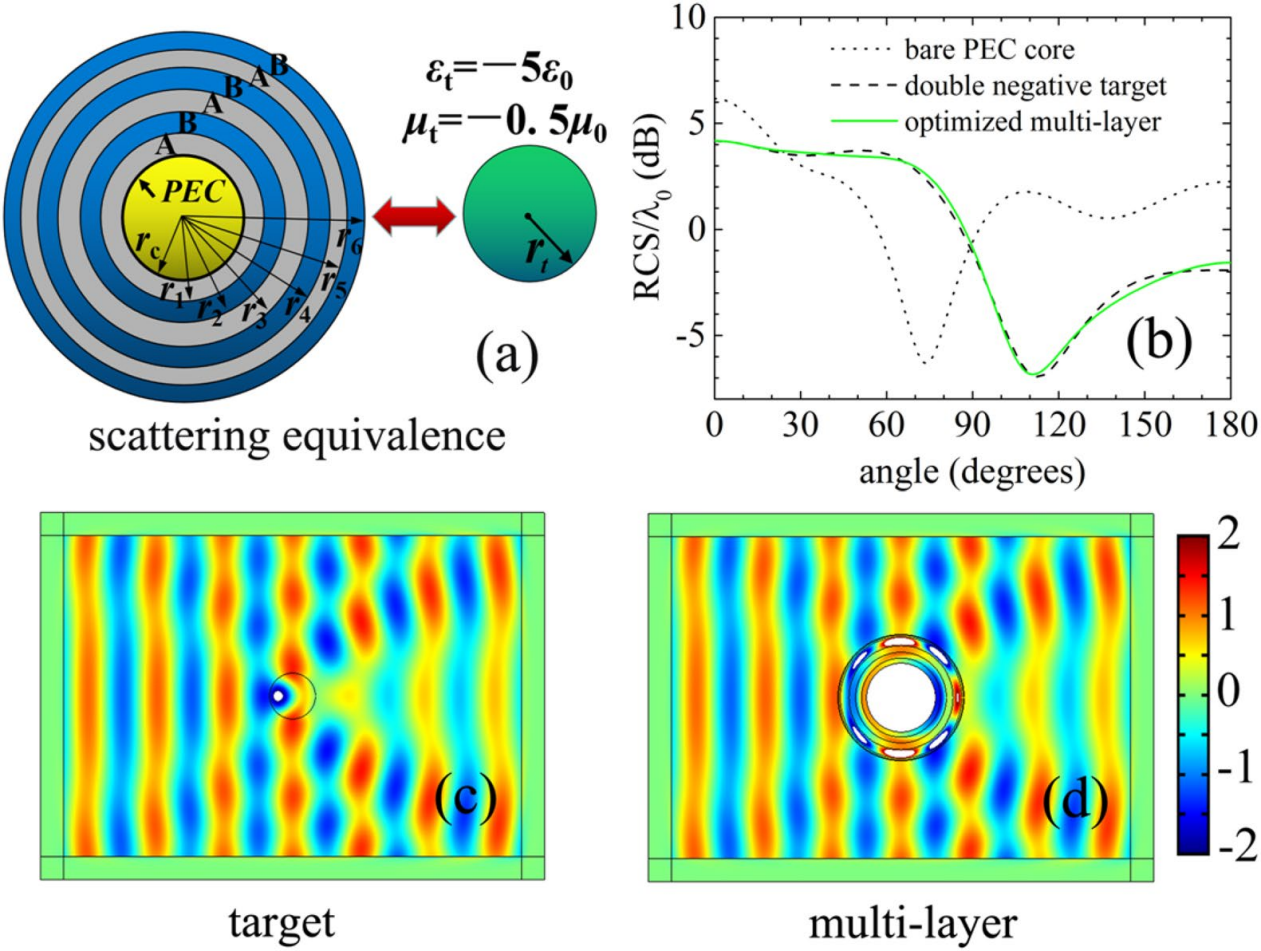
**(a)** Schematic diagram of scattering equivalence. A PEC core (*r*_*c*_ = λ_0_**/**2) covered by an optimized 6-layer shell can be disguised as a target with double negative material (*r*_*t*_ = λ_0_**/**3, *ε*_*t*_ = -5*ε*_0_, *μ*_*t*_ = -0.5*μ*_0_). **(b)** The scattering widths normalized by wavelength for the bare PEC core (dotted black line), the double negative target (dashed black line), and the optimized design (solid green line). The magnetic field distributions of the double-negative-material target **(c)**, and the optimized 6-layer illusion device **(d)** are simulated using COMSOL Multiphysics. The magnitudes out of the colour bar range are represented by white area.

Finally, a conducting cylinder with radius *r*_*c*_ = λ_0_**/**2 is considered. We want to make the conducting cylinder behave like a double-negative-material target with *r*_*t*_ = λ_0_**/**3, *ε*_*t*_ = −5*ε*_0_ and *μ*_*t*_ = −0.5*μ*_0_, shown in Fig. 4a. A 6-layer shell composed of two alternating nonmagnetic materials A and B is constructed to realize the scattering equivalence. Material A is an epsilon-near-zero **(**ENZ) medium with permittivity *ε*_*A*_ = 0.1*ε*_0_, and Material B is the commonly used material silica with permittivity *ε*_*B*_ = 12.08*ε*_0_. As reported in our previous research of optimized invisibility cloak, this multi-layered configuration with two alternating materials of large dielectric constants difference can have good scattering manipulation ability^26^. The outer radius of each layer is optimized within an appropriate range to ensure a moderate size, and the optimized results of the six layers by GA are as follows: *r*_1_ = 0.5728λ_0_, *r*_2_ = 0.6587λ_0_, *r*_3_ = 0.7551λ_0_, *r*_4_ = 0.8818λ_0_, *r*_5_ = 0.905λ_0_ and *r*_6_ = 0.9166λ_0_. Figure 4b shows the bistatic RCSs of the bare PEC core, the double negative target and the optimized design. It can be seen that the RCS of the optimized multi-layer at all angles is approximately equal to that of the target. Figure 4c,d show the total magnetic field simulations of the target and the optimized design. Nearly the same distributions in the exterior domain verify the effectiveness of the illusion effect based on the ENZ material.

## Discussion

**Figure 5:**
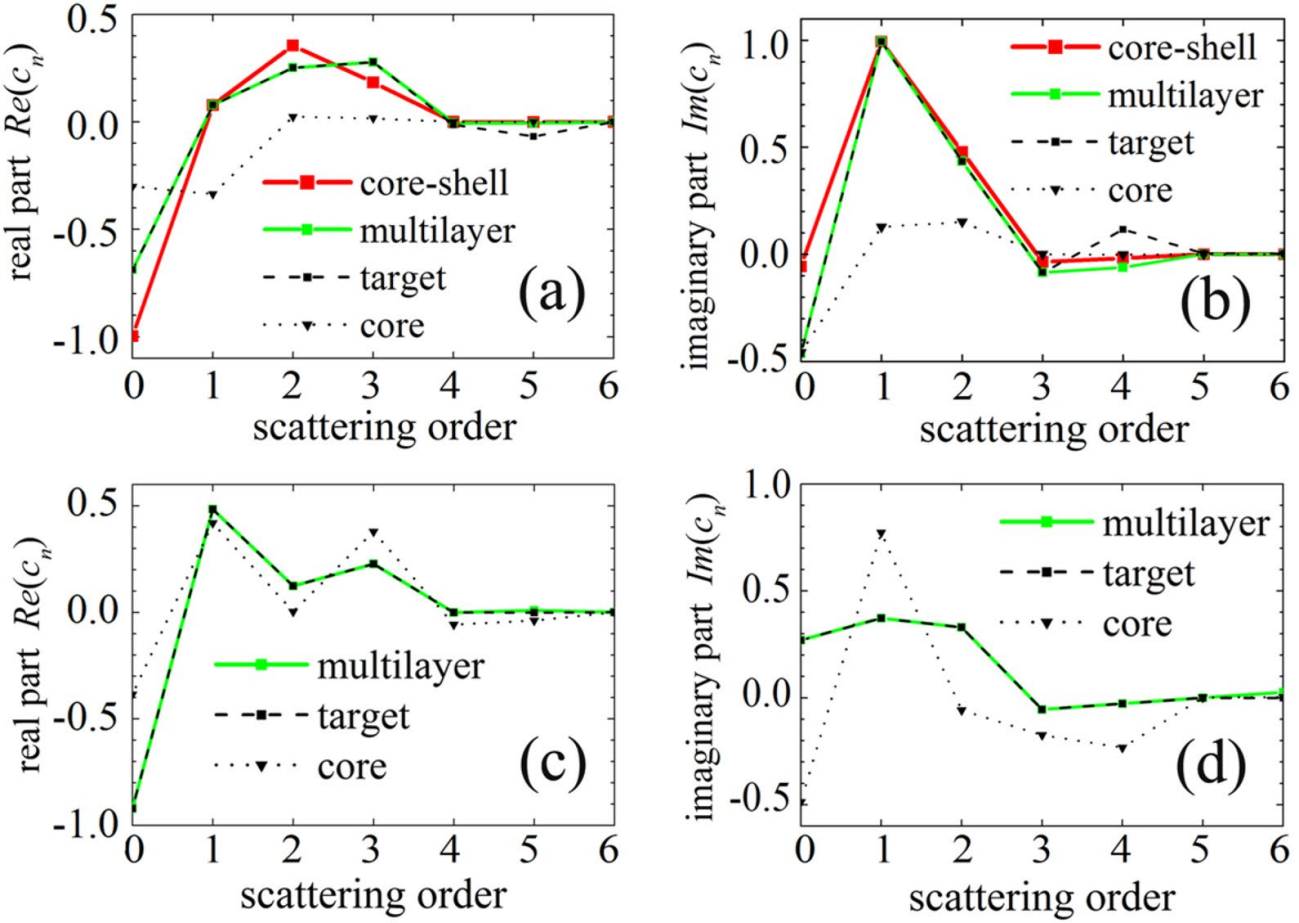
Real part **(a)** and imaginary part **(b)** of the Mie scattering coefficients for the core cylinder (*r*_*c*_ = λ_0_**/**4, *ε*_c_ = 2*ε*_0_, *μ*_c_ = *μ*_0_), the target (*r*_*t*_ = λ_0_**/**2, *ε*_*t*_ = 6*ε*_0_, *μ*_*t*_ = *μ*_0_), the optimized core–shell illusion device, and the optimized 3-layer device. Real part **(c)** and imaginary part **(d)** of the scattering coefficients for the PEC core (*r*_*c*_ = λ_0_**/**2), the double-negative-material target (*r*_*t*_ = λ_0_**/**3, *ε*_*t*_ = −5*ε*_0_, *μ*_*t*_ = −0.5*μ*_0_), and the optimized 6-layer illusion device.

The reason that quasi-perfect illusion effect is achieved with only a few optimized layers can be explained as follows. In the GA, the fitness function denoted by Eq. (3) is determined by the difference between the scattering coefficients of the multi-layered illusion device and the target. These two kinds of scattering coefficients may be close to each other after optimization. To demonstrate this, the real and imaginary parts of the coefficients for the above two optimized examples are plotted in Fig. 5. Figure 5a,b show that the first six coefficients *c*_*n*_ (*n* = 0 ~ 5) of the target with parameters (*r*_*t*_ = λ_0_**/**2, *ε*_*t*_ = 6*ε*_0_, *μ*_*t*_ = *μ*_0_) are dominant and other higher-order coefficients can be negligible. Although the coefficients of the core are quite different from that of the target, the curve of the optimized multi-layer almost coincides with that of the target, except for *Re*(*c*_5_) and *Im*(*c*_4_). The first five coefficients (*n* = 0 ~ 4) of the core–shell structure deviate a little from that of the target, except for the dipolar coefficient (*n* = 1). Therefore, compared with the core–shell design, the optimized 3-layer illusion device has more optimized parameters to achieve a better illusion effect. Furthermore, Fig. 5c,d show that the scattering coefficients of the optimized six-layer illusion device and the double-negative-material target are almost the same. In a word, the physical mechanism for the multi-layered illusion device is that superposition of the multi-polar moment of the same order in the core and the multi-layered shell is equal to that of the target. When we consider a very small target from which the scattering is dominated by the dipole mode, the mechanism of the core–shell illusion device can be easily explained by the conclusion that the total dipole moments in the core and the covering shell are equivalent to that of the target.

## Conclusion

In conclusion, we have studied the optimized design for the illusion device with isotropic and homogeneous materials. For an electrically small target, a core–shell structure with the optimized material and geometrical parameters can achieve better illusion effect than the analytical design based on the scattering cancellation. For a target with dimension comparable to the wavelength, we have presented two optimized examples: one is to make a dielectric object appear as another dielectric target, and the other is to make a PEC cylinder behave like a double-negative-material target. Therefore, it is concluded that the optimized design method can greatly widen the application range of the illusion device. In comparison, the transformation-based illusion devices usually use very complex materials, and the scattering-cancellation illusion devices are preferable for objects with small dimension. Our proposed multi-layered illusion devices take both the advantages of these two methods: (1) the transformation-based illusion devices are applicable to large objects;

(2) the scattering-cancellation illusion devices adopt isotropic and homogeneous materials. Our optimized method can also be applied to the design of other novel electromagnetic devices or extended to other physical fields, such as acoustics and thermodynamics.

## Methods

In this paper, genetic algorithm (GA) is used to optimize the cylindrical multi-layered illusion device. GA is an optimization method to search for optimal solution by simulating natural evolution process, which can be applied to solve a wide range of real-world problems of significant complexity, including the problems of discontinuous, non-differentiable, and highly nonlinear objective function^28^. Here, we utilize the GA toolbox in the MATLAB software to implement the optimization process, which is easy to set the range of optimized parameters considered from the practical situation. GA manipulates a population of artificial chromosomes which are string representations of solutions to the problem. Each chromosome has a fitness that measures how well the problem is solved. Starting with a randomly generated population of chromosomes, the GA randomly selects individuals from the current population as parents and carries out a process of fitness-based selection and recombination to produce children for the next generation. After several generations, the population evolves to the optimal solution. For the optimized illusion device of the cylindrical multi-layered structure, GA is used to minimize the fitness function by searching the optimal permittivities and the thicknesses of the covering layers.
